# Mg Biodegradation Mechanism Deduced from the Local Surface Environment under Simulated Physiological Conditions

**DOI:** 10.1002/adhm.202100053

**Published:** 2021-05-29

**Authors:** Jorge Gonzalez, Sviatlana V. Lamaka, Di Mei, Nico Scharnagl, Frank Feyerabend, Mikhail L. Zheludkevich, Regine Willumeit‐Römer

**Affiliations:** ^1^ Institute of Metallic Biomaterials Helmholtz‐Zentrum Hereon Geesthacht 21502 Germany; ^2^ Institute of Surface Science Helmholtz‐Zentrum Hereon Geesthacht 21502 Germany; ^3^ School of Materials Science and Engineering & Henan Key Laboratory of Advanced Magnesium Alloy Zhengzhou University Zhengzhou 450001 P. R. China; ^4^ Institute for Materials Science Faculty of Engineering Kiel University Kiel D‐24143 Germany

**Keywords:** biodegradable materials, biometals, implants, interfaces, local pH, magnesium, SIET

## Abstract

Although certified magnesium‐based implants are launched some years ago, the not well‐defined Mg degradation mechanism under physiological conditions makes it difficult to standardize its use as a degradable biomaterial for a wide range of implant applications. Among other variables influencing the Mg degradation mechanism, monitoring the pH in the corrosive solution and, especially, at the corroding interface is important due to its direct relation with the formation and stability of the degradation products layer. The interface pH (pH at the Mg/solution interface) developed on Mg‐2Ag and E11 alloys are studied in situ during immersion under dynamic conditions (1.5 mL min^‐1^) in HBSS with and without the physiological amount of Ca^2+^ cations (2.5 × 10^‐3^ m). The results show that the precipitation/dissolution of amorphous phosphate‐containing phases, that can be associated with apatitic calcium‐phosphates Ca_10‐_ *_x_* (PO_4_)_6‐_ *_x_* (HPO_4_ or CO_3_)*_x_* (OH or ½ CO_3_)_2‐_ *_x_* with 0 ≤ *x* ≤ 2 (Ap‐CaP), promoted in the presence of Ca^2+^ generates an effective local pH buffering system at the surface. Thus, high alkalinization is prevented, and the interface pH is stabilized in the range of 7.6 to 8.5.

## Graphical abstract

In the present work, the local interface pH, the degradation rate, and the degradation products' layer's characterization of two different magnesium alloys are correlated. The precipitation/dissolution processes forming the different degradation products layer are found to act as a local surface buffering system that can effectively buffer differences in alkalinization rates coming from significant differences in the degradation rate.

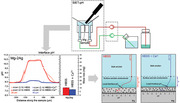

## 1 Introduction

In contrast with traditional biologically inert implant materials, degradable biomaterials emerged aiming a controlled in vivo degradation, avoiding the need for a second surgery to remove the implant. Within degradable biomaterials, some metals (Mg, Zn, Fe) are advantageous given their mechanical and biocompatibility properties. Mg‐based materials^[^ ^1^, ^2^, ^3^ ^]^ are the current leading‐edge biodegradable metals for bone remodeling. However, despite the introduction of some Mg‐based implants for bone fixation (MAGNEZIX and Resomet) and atherosclerosis treatments (Magmaris), some questions remain unclear from a fundamental point of view, and that hinders their introduction for new applications: i) a current mismatch between mechanical properties for high‐load applications and acceptable degradation rates, ii) the unclear physiological paths of released Mg^2+^ on the complex bone remodeling process^[^ ^4^ ^]^ and the response of other dissolution products including its possible physiological accumulation (e.g., alloying elements and H_2_) in the tissue healing process,^[^ ^5^ ^]^ iii) the complex and not the well‐defined physiological environment of different implantation sites hinders the possibility of direct laboratory mimicking of the in vivo conditions^[^ ^6^, ^7^ ^]^ and difficult to understand the degradation mechanism.

A well‐established degradation mechanism under physiological conditions should describe which chemical and electrochemical reactions take place at the interface leading to the formation of the degradation products layer and how those are influenced by; i) the physiological fluid components (inorganic, organic components) and cell presence, ii) the fluid exchange, iii) the composition and microstructure of the alloy, and iv) the mechanical solicitation (static or dynamic loads). The composition and morphology of the degradation products layer have a crucial effect on the degradation rate and degradation morphology of Mg alloys. Due to the complexity of the physiological fluids and the not well‐understood individual effects of their components on Mg‐based degradation mechanisms, current studies observe simplified in vitro degradation conditions. On the other hand, besides the complexity introduced into the degradation mechanisms by the organic fraction of physiological fluids (e.g., proteins,^[^ ^8^, ^9^ ^]^ glucose and amino acids,^[^ ^10^ ^]^ vitamins,^[^ ^11^ ^]^ and the presence of cells^[^ ^12^, ^13^ ^]^), the apparently simple inorganic fraction of physiological fluids plays a crucial role, which is still not fully understood. First of all, the presence of physiologically relevant ions Ca^2+^, HPO_4_^2–^ or HCO_3_^–^, revealed a critical influence on the degradation rate of Mg alloys.^[^ ^6^, ^7^, ^14^ ^]^ The interaction of those inorganic components within the implant surface environment rules the implant material's initial contact with the physiological solution affecting the subsequent interaction with the different organic components.^[^ ^15^ ^]^ Moreover, previous works revealed how proteins,^[^ ^9^, ^16^ ^]^ glucose,^[^ ^10^, ^17^ ^]^ and amino acids,^[^ ^9^ ^]^ and the presence of osteoblast^[^ ^12^, ^13^ ^]^ generates a higher amount of protective apatite‐like calcium phosphate phases (Ap‐CaP) phases in the degradation products layer.

Heterogeneous precipitation–dissolution equilibria between the inorganic ions in solution and the precipitated phases at the metallic surface ruled the inorganic compounds’ presence in the degradation products layer. Those equilibriums are conditioned by; i) the thermodynamic concept of supersaturation and the kinetic conditions of ii) nucleation, iii) crystal growth, and iv) the agglomeration.^[^ ^18^ ^]^ The kinetic conditions are challenging to assess, especially under the complexity of the simulated physiological conditions; therefore, previous works^[^ ^7^, ^19^ ^]^ focus on the calculation of the supersaturation to justify the presence of inorganic phases in the degradation product layer according to the temperature and the ionic activities of the bulk degradation solution. However, the limitations to the mass transfer between the reactive magnesium surface and the bulk solution,^[^ ^20^ ^]^ which are defined by a specific in vitro set‐up or implantation site environment, can promote significant differences between the ionic environment in the bulk solution and the solution near the surface. This fact leads to the need for localized measurements of ionic concentrations that can monitor changes both within the bulk solution and in the solution near the magnesium surface.

Moreover, due to the basic character of anions present in compounds found in the degradation products layer (e.g., OH^–^, CO_3_^2–^, PO_4_^3–^), the heterogeneous precipitation equilibria might be affected by the solution pH through a secondary acid–base reaction, according to Le Chatelier´s principle.^[^ ^21^ ^]^ Scanning electrochemical microscopy (SECM)^[^ ^22^ ^]^ are a group of techniques capable of mapping specific chemical species in liquid environments with a high spatial resolution. After been widely used in biological studies in the past three decades, only recently studies provided information on the solid/liquid interfaces processes at a microscale. The perpendicular pH profiles to the Mg–Ca system surface by SECM performed by Mareci et al.^[^ ^23^ ^]^ recently confirmed differences between the pH within the bulk solution and the pH at the surface. However, the absence of a buffering system, the absence of relevant physiological ions in the degradation solution, and the semi‐static set‐up adopted might be the reason for changes of the pH profiles with the immersion time and the alloy composition. The in situ SECM mapping of the AZ31 alloy reported by Jamali et al.^[^ ^24^ ^]^ in SBF containing HEPES as buffering agent showed 11.9 pH units in the highly active zones and 9.6–9.8 pH units for the majority of the analyzed surface. However, the 195 µm tip diameter of the Ir/IrOx probe, the potential dependency of the Ir/IrOx probe to the local concentration of O_2_ and H_2,_^[^ ^25^ ^]^ the capacity of HEPES to alter the degradation under physiological conditions,^[^ ^8^, ^26^ ^]^ and the static conditions of the set‐up could promote higher interface pH values. To understand the above discrepancies and correlate the interface pH to the alloy performance, our previous study^[^ ^27^ ^]^ applied a scanning ion‐selective technique (SIET) testing set‐up to measure in situ the interface pH. For those measurements, different Mg alloys were immersed in Hank´s balanced salt solution (HBSS) under a flow rate of 1.5 mL min^‐1^ to mimic the physiological fluid composition homeostasis better and improve the pH buffering capacity. Under those conditions, the different composition and microstructures of the Mg alloys developed similar and stable interface and bulk pH values. An interface pH between 10 and 11 units, the typical value for degradation of Mg alloys in simple NaCl solutions,^[^ ^28^, ^29^, ^30^ ^]^ and higher degradation rates were revealed for under immersion in HBSS in the absence of Ca^2+^ cations. In turn, an interface pH between 7.8 and 8.5 pH units and significantly lower degradation rates were found with the presence of Ca^2+^ cations. In a subsequent work,^[^ ^31^ ^]^ we identified the protective effect of the presence of Ca^2+^ with a synergic effect between the Ca^2+^, Mg^2+^, HCO_3_^–^ and HPO_4_^2–^ ions that promote the formation of an additional protective layer growing beside the inner MgO/Mg(OH)_2_ layer.

The present article aims to explain the interface pH and the degradation rate values by characterizing the degradation product layer. For this target, two Mg alloys with substantial differences in degradation rates (extruded Mg‐2Ag and as‐cast E11) were immersed in simulated body fluids based on HBSS varying the presence of Ca^2+^, Mg^2+^, HCO_3_^–^ and H_2_PO_4_^–^/HPO_4_^2–^ ions and characterizing the degradation products layer generated.

## 2 Experimental Section

### 2.1 Magnesium Alloys

Two different Mg alloy systems recently revealed as candidates for degradable biomaterials, Mg–Ag^[^ ^32^, ^33^, ^34^ ^]^ and Mg‐Nd‐Gd‐Ca,^[^ ^35^, ^36^, ^37^ ^]^ were used in the present work. As‐cast E11 (Mg‐Gd‐Nd‐Ca) and extruded and T4 heat‐treated Mg‐2Ag, produced at Magnesium Innovation Center (MagIC), Helmholtz‐Zentrum Hereon. According to the data presented in previous work,^[^ ^27^ ^]^ both alloys’ chemical composition is presented in **Table** **1**.

**Table 1:** Chemical composition in wt% of tested Mg alloys

	Ag^a)^	Al	Ca^b)^	Cu	Fe	Gd^c)^	La	Mn	Ni	Nd	Si	Zn	Zr	Pr	Mg
Mg–2Ag	1.99	0.034	–	0.0013	0.0027	n/a	–	–	0.0035	–	–	n/a	n/a	–	Balance
E11	–	0.037	0.19	0.0049	0.0015	9.25	0.0134	0.0462	>0.018	1.36	0.0008	0.0053	0.0022	0.33	Balance

^a)^ Ag: AAS, Agilent Technologies 240FS AA, *λ* = 328.1 nm;

^b)^ Ca: AAS, Agilent Technologies 240FS, Matrix standardized method (Gd, Nd, Mg), *λ* = 422.7 nm;

^c)^ Gd: μXRF M4 Tornado (Bruker), 25 µm spot analysis (Mo K), 20 × 20 mm area analyzed, 8 ms/pixel.

The as‐cast E11 alloy was directly machined into Ø 10 × 1.5 mm discs. The as‐cast Mg‐2Ag alloy was homogenized at 450 °C for at least 8 h. Afterwards, the alloy was quenched by dipping into a cold‐water bath. The resultant homogenized ingot was machined into a Ø 100 × 200 mm cylinder for the extrusion process, which was subsequently processed by hot indirect extrusion up to 9 mm diameter rods. The rods were cooled down under atmospheric conditions, heat‐treated under T4 conditions, quenched in water, and machined into Ø 9 × 1.5 mm discs.

### 2.2 Metallographic Preparation

The samples were embedded in Demotec 30 (Demotec metallographic, Nidderau, Germany) methyl methacrylate‐based resin to reveal the alloys’ microstructure and the morphology and composition of the degradation products layer. After the resin solidification, the surface was ground with SiC paper down to P2500. Then the samples were polished in the presence of water‐free oxide polishing suspension (OPS). The residual OPS and ground material was removed with 100% ethanol in an ultrasonic bath, and the samples were analyzed by optical microscopy and scanning electron microscopy. To reveal the grain size and microstructure distribution, the polished surfaces were etched for a few seconds in a picric acid solution dissolved in water (17%), ethanol (79%), glacial acetic acid (4%, all chemicals by VWR International). After the etching, the samples were rinsed in 100% ethanol and dried by compressed hot air.

### 2.3 Local pH Measurements by Scanning Ion‐Selective Electrode Technique (SIET)

**Figure 1:**
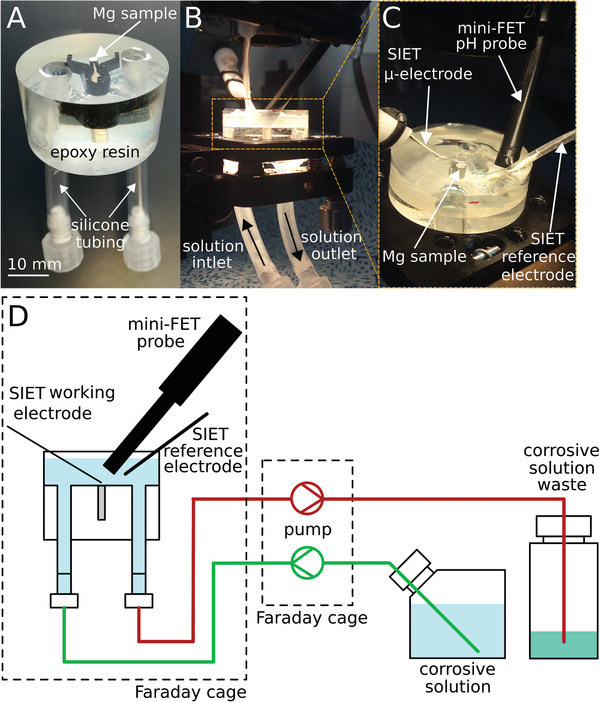
a) General description of the set‐up used in the in situ interface pH measurements. b,c) details of the Mg sample assembling in the SIET set‐up. d) Scheme of the dynamic conditions applied during the interface pH measurements by SIET.

Two samples of the previously described alloys were machined up to approximately 1.5 mm (Mg‐2Ag) and 2.5 mm (E11) diameter. The samples were cleaned with 100% ethanol in an ultrasonic bath and embedded in epoxy resin, configuring a homemade flow cell with approximately a 5 mL volume, as described in **Figure** **1**.

The samples were polished with 3 µm diamond suspension and then in a mixture of 0.25 µm OPS water‐free suspension to generate a surface free of imperfections that allowed the microelectrode's free movement 10–40 µm above the surface. A repolishing process was the only necessary procedure for reusing the embedded sample in subsequent experiments. Thus, multiple consecutive experiments were conducted on the same physical sample that ensures comparability of the results. pH‐sensitive glass‐capillary microelectrodes with a tip orifice of 2 µm, were made of salinized glass‐capillaries by backfilling them with a solution of 0.01 m KH_2_PO_4_ in 0.1 m KCl and tip filling them with ionophore‐based membrane selective for H^+^ (Fluka, Ref. 95293).^[^ ^27^ ^]^ A homemade silver–chloride Ag/AgCl/0.1 m KCl, 0.01 m KH_2_PO_4_ electrode was an external reference electrode. The probes were positioned 10 to 40 ± 5 µm above the sample surface for parallel SIET scans, and 500‐points line scans were performed by a point‐by‐point, move‐wait (0.6 s)‐measure (0.8 s) routine, which took about 40 min to complete. The in situ interface pH measurements were performed under flow conditions with a commercial SVET/SIET/DVIT system from Applicable Electronics controlled LV4 software (ScienceWare, New Haven, USA), according to the measuring procedure exposed in previous work.^[^ ^27^ ^]^ A TL15E peristaltic pump (Medorex, Nörten‐Hardenberg, Germany) was applied to induce a pulsatile flow rate of 1.5 mL min^‐1^ of the electrolyte. The flow was distributed by Versilic silicone tubes (Saint Gobain, IDEX Health & Science GmbH, Wertheim, Germany) and Norpren A‐60‐G tubes (Saint Gobain, IDEX Health & Science GmbH, Wertheim, Germany) used for the pumping process. As shown in Figure 1, the set‐up was permanently open to the atmosphere, therefore equilibrated with the air. The flow‐through set‐up was cleaned for 30 min in demineralized water under an ultrasound bath and disinfected by pumping 70% ethanol for 30 min. Before every test after the cleaning and sterilization process, the system was rinsed with HBSS.

### 2.4 Dynamic Degradation Tests

In order to analyze the degradation products layer composition and morphology, one disc of both alloys (Section 2.1) was immersed (between 10 min and 3 h), under dynamic conditions (1.5 mL min^‐1^), in the different simulated physiological solutions presented in **Table** **2**. Before the immersion, the samples were ground with SiC paper down to P2500 and then cleaned with 100% ethanol in an ultrasonic bath. As in the SIET set‐up, the degradation chamber was also permanently open to the atmosphere.

**Table 2:** Dissociated composition in ×10^‐3^ m of the degradation solutions applied in the immersion tests performed in this work and compared with the blood plasma and the Interstitial fluid composition (Note: The values in brackets correspond to the ionic concentrations not bound to proteins)

Solution	Na^+^	Mg^2+^	Cl^–^	K^+^	Ca^2+^	SO_4_^2–^	HCO_3_^–^	HPO_4_^2–^	d‐glucose
Blood plasma^[^ ^8^, ^38^, ^39^, ^40^, ^41^, ^42^, ^43^, ^44^ ^]^	142	1.5 (1.0)	103	5.0	2.5 (1.3)	0.5	22‐30	1.0	3.6‐5.2
Interstitial fluid^[^ ^39^, ^43^, ^45^ ^]^	136–146	0.5– 0.7	115	4.0	1.2–1.5	0.7	26	0.6–1.7	1.0
HBSS‐based media used in this work									
I (ref. 14175053)	142.8	–	143.3	5.8	–	–	4.2	0.8	5.5
II	138.6	–	148.3	5.8	2.5	–	–	0.8	5.5
III	137.9	–	148.2	5.3	2.5	–	–	–	5.5
IV	142.1	–	148.2	5.3	2.5	–	4.2	–	5.5
V	138.5	–	148.2	5.7	2.5	–	–	0.8	5.5

### 2.5 Electrolytes for Degradation Tests

The alloys were tested in solutions contacting physiological electrolytes based on Hank´s balanced salt solution (HBSS) at room temperature and during different immersion times. The detailed composition of the solutions compared with different body fluids is presented in Table 1. The compositional additions or reconstructions of the commercial HBSS (ThermoFisher Scientific, ref. 14175053) were performed with the following chemicals in an analytical degree; NaCl (Sodium chloride ≥ 99%, Sigma Aldrich), KCl (Potassium chloride, ≥99.5% Merck, Darmstadt, Germany), CaCl_2_·2H_2_O (Calcium chloride dihydrate, ≥ 99% Fluka, Germany), NaHCO_3_ (Sodium hydrogen carbonate, ≥99.5%, Merck), Na_2_HPO_4_·2H_2_O (di‐sodium hydrogen phosphate, ≥ 99.5%, Merck), KH_2_PO_4_ (potassium dihydrogen phosphate, ≥ 99.5% Merck), C_6_H_12_O_6_ (D(+)‐glucose, >99%, Fluka BioChemika).

### 2.6 Micro‐X‐Ray Fluorescence (μXRF)

A 2D energy dispersive (EDXRF) X‐ray spectrometer μXRF M4 TORNADO (Bruker, Ettlingen, Germany) was used for elemental analysis and relative quantification of the degradation products layer. The X‐ray Rhodium (Rh) anode, set for 50 kV and 600 µA, and the poly‐capillary optics generate a spot size down to 25 µm. The measurements were conducted under vacuum at 20 mbar. A 12.5 µm aluminium foil filter was applied to cut off the scattered source excitation and reduce the diffraction peaks of possible crystalline materials. The average spectra of 3 cycles over a sample area of 1.5 cm^2^ were analyzed with a 25 µm distance between analyzed points.

### 2.7 Grazing Angle X‐Ray Diffraction (GAXRD)

The corroded samples’ surface was analyzed by a Bruker D8 Advance (Bruker, Karlsruhe, Germany) in a grazing incidence geometry of 1 degree. The generator was set for 40 kV and 40 mA, and the data were collected between 15 and 80 degrees of 2*θ* at intervals of 0.02°. The cutting time was 2 s per data point. The data analysis was carried out by BrukerEVA and PDF‐2 (Release 2015 RDB) analysis software.

### 2.8 Fourier Transform Infrared Spectroscopy Attenuated Total Reflectance (FTIR‐ATR)

The corroded samples’ surface was rinsed in 100% ethanol and stored under vacuum until analyzed by an FTIR‐ATR equipment Tensor 27 (Bruker, Ettlingen, Germany) to measure the IR spectra of the degradation products formed on the surface. Every spectrum is the result of an average of 512 scans with a resolution of 2 cm^–1^. The data evaluation was performed by OPUS software version 7.5.18 (Bruker, Ettlingen, Germany).

### 2.9 Solubility Modeling

The Medusa‐Hydra chemical equilibrium modeling software developed by Puigdomenech^[^ ^46^ ^]^ was used to correlate the thermodynamic stability of the possible compounds present in the degradation products layer with the conditions presented during the immersion tests (simulated physiological solution composition and interface pH).

### 2.10 Electrochemical Impedance Spectroscopy

For the electrochemical impedance spectroscopy (EIS) measurements, a conventional three‐electrode set‐up was used, including a working electrode (exposure area of 0.5 cm^2^, electrolyte volume of 330 mL), a Pt wire coil counter electrode and a saturated Ag/AgCl reference electrode. The samples were ground to 1200 grit with SiC paper before measurement, cleaned with ethanol and dried by a pressurized air stream. EIS measurements were performed at open circuit potential (OCP), applying a sinusoidal perturbation with an amplitude of 10 mV RMS over a frequency range from 100 kHz to 0.1 Hz. EIS was measured until 0,1 Hz to avoid the possible polarization caused by potential non‐stationarities during the long measurements at low frequencies. In this case, fast scans can be performed during the initial minutes of tests. The EIS spectra were recorded using a Gamry Interface 1000 potentiostat/galvanostat under constant steering condition (200 r.p.m.) at 22 ± 2 °C.

## 3 Results

### 3.1 Alloys´ Microstructure

**Figure 2:**
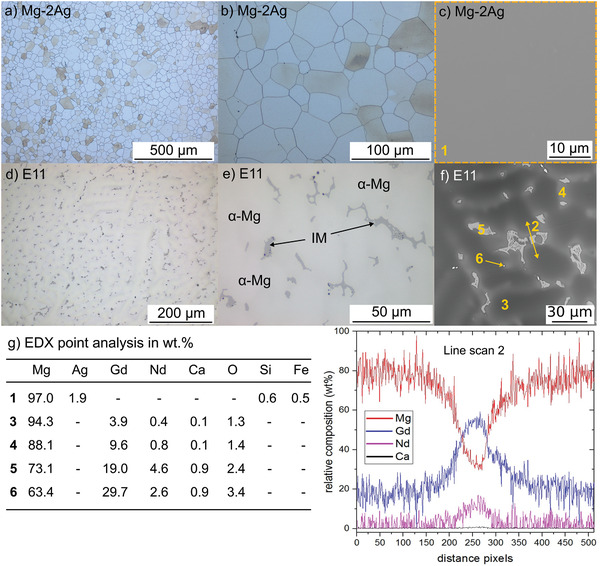
Alloy microstructure evaluated by OM and BSE‐SEM of the a–c) extruded and T4 heat‐treated Mg–2Ag alloy, and d–f) as‐cast E11 alloy. The correspondent relative chemical analysis performed by EDX of the different areas of the microstructures is presented in (g,h). [IM: Intermetallic phase]

The different microstructures of the extruded Mg‐2Ag(T4) and the as‐cast E11 alloys are shown in **Figure** **2**.

Figure 2a,b shows the Mg‐2Ag alloy, an 25 to 15 µm diameter equiaxial grain microstructure of the *α*‐Mg matrix phase. The BSE image of Figure 2c and the EDX analysis 1 in Figure 2g show a homogeneous surface that points out the total solubilization of the amount of Ag in the *α*‐Mg. Under the as‐cast condition of the E11 alloy, Figure 2d–f shows a dendritic as‐cast microstructure formed by the primary *α*‐Mg dendrites with Gd, Nd and Ca in solid solution, as confirmed by the EDX analysis 3 in Figure 2g. A well‐connected cellular network of Gd, Nd‐segregated *α*‐Mg surrounds the primary *α*‐Mg dendrites matrix, revealed as a slightly brighter phase than the primary *α*‐Mg by BSEM and the EDX analysis 4 in Figure 2g. This segregated *α*‐Mg surround a dense but not well‐connected network of a Gd, Nd‐rich bright intermetallic secondary phase (IM), according to the EDX analysis 5 in Figure 2g, with a pseudo‐eutectic morphology and probably composed of *β*‐Mg_5_(Gd, Nd), as was identified previously.^[^ ^35^, ^47^ ^]^ This IM phase, in turn, encloses even brighter cuboidal particles between 0.5 and 5 µm diameter, with higher content in Gd and Nd as revealed in the EDX analysis 6 in Figure 2g, that were identified previously as [Gd, Nd, Mg]‐hydrides (REH2/REH3).^[^ ^48^, ^49^ ^]^

### 3.2 Ca^2+^ Cations Presence and Interface pH

**Figure 3:**
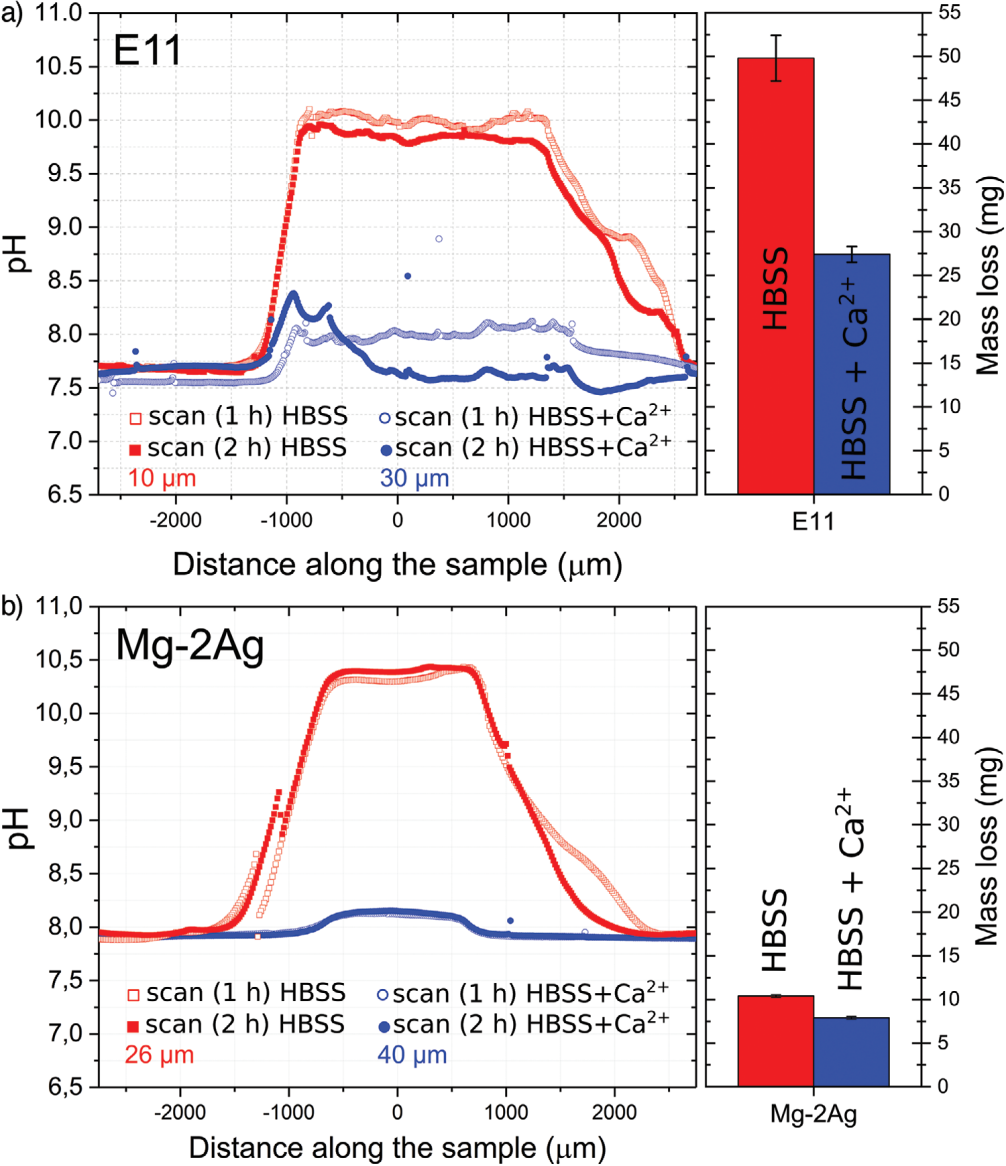
Influence of the presence of Ca^2+^ cations in the HBSS solution composition on the interface pH along the two first hours of dynamic immersion (1.5 mL min^‐1^), and the mass loss after 24 h of dynamic immersion (1.5 mL min^‐1^) for the a) E11 alloy and b) Mg–2Ag alloy.

The influence of the Ca^2+^ cations on the in situ interface pH is revealed in **Figure** **3**a,b by comparing the results of interface pH under immersion of the E11 and Mg‐2Ag samples in HBSS and HBSS with the presence of Ca^2+^ cations. The results present two consecutive parallel scans performed between 30 and 40 µm above the surface during the first 119 min of dynamic immersion. The immersion in HBSS revealed an interface pH for the E11 alloy between 9.8 and 10.1 units at 10 µm above the surface, while for the Mg–2Ag alloy, the interface pH was between 10.2 and 10.4 units at 26 µm above the surface. On the contrary, the addition of 2.5 × 10^‐3^ m of CaCl_2_.2H_2_O to the HBSS composition generated an interface pH between 7.6 and 8.1 units for the E11 alloy and around 8.1 units in the case of the Mg–2Ag alloy. A similar difference in the interface pH values in HBSS electrolyte with/without Ca^2+^ was discovered for two more Mg alloys in our previous work.^[^ ^27^ ^]^ Moreover, the presence of Ca^2+^ cations in the HBSS composition revealed a decrease in the degradation rate shown by the mass loss measurements in Figure 3.

Due to the as‐cast status of the E11 alloy, the content of noble alloying elements (Gd, Nd), and the high content of impurities, the protective effect generated by the presence of Ca^2+^ cations is significantly stronger than the one generated for the Mg–2Ag alloy. Ca‐containing phases justify the similar low interface pH developed in the presence of Ca^2+^ cations for different Mg alloys with big differences in the mass loss in the degradation product layer. Those phases increase the degradation products layer's passivation capacity significantly; however, this protective effect can only modulate the intrinsic degradation rate and, therefore, still reveal differences between Mg alloys. On the other side, the precipitation‐dissolution processes of those Ca‐containing phases generate an extra buffering system at the interface that maintains the interface pH around 8 units even for Mg alloys that show strong differences in mass loss.

**Figure 4:**
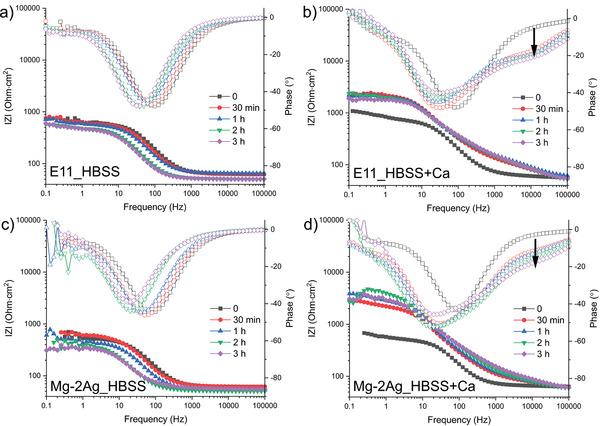
Bode plots for the E11 (up) and Mg–2Ag (down) alloys under immersion a,c) HBSS composition and b,d) under HBSS with CaCl_2_·2H_2_O, between 0 and 3 h of immersion.

The protective capacities of the degradation products layer formed in the absence and presence of Ca^2+^ cations were studied by EIS and presented in **Figure** **4**. The evolution of Bode plots over 3 h of immersion shows a similar trend for E11 and Mg–2Ag. The appearance of an additional time constant at the high‐frequency range (marked by an arrow in Figure 4b–d) for the immersion of E11 and Mg‐2Ag in Ca^2+^‐containing HBSS indicates the formation of an additional protective layer. In previous work, this additional layer's formation has been proven to be mainly controlled by the inorganic ions in the medium rather than the substrate.^[^ ^11^ ^]^ Figure 4 also shows a marked increase of the low‐frequency impedance for both alloys exposed to Ca^2+^ containing HBSS compared to that in simple HBSS.

### 3.3 Characterization of Degradation Products Layer

The influence of the presence of the Ca^2+^ cations on the degradation morphology and the degradation products composition generated after 3 h of dynamic immersion of E11 and Mg–2Ag alloys was evaluated by SEM with backscattering and EDX detectors, μXRF, GAXRD and FTIR‐ATR.

**Figure 5:**
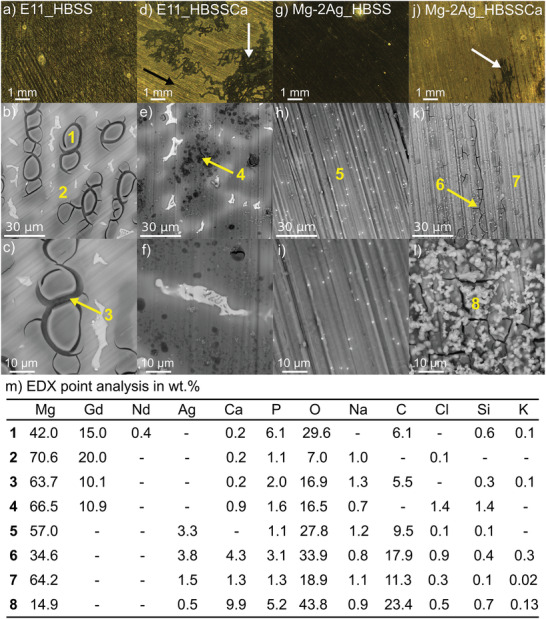
Analysis of the changes induced by the degradation process on the surface aspect (by OM) and microstructure (by BSE‐SEM) after 3 h of dynamic immersion (1.5 mL min^‐1^) of the E11 surface in (a– c) HBSS (solution I) and (d–f) HBSS with the presence of 2.5 × 10^‐3^ m of Ca^2+^ cations (solution III), and of the Mg–2Ag surface in (g–i) HBSS (solution I) and (j–l) HBSS with the presence of 2.5 × 10^‐3^ m of Ca^2+^ cations (solution III). The relative chemical analysis in wt% performed by EDX in the denoted points at the images a–l are detailed in the table m.

**Figure 6:**
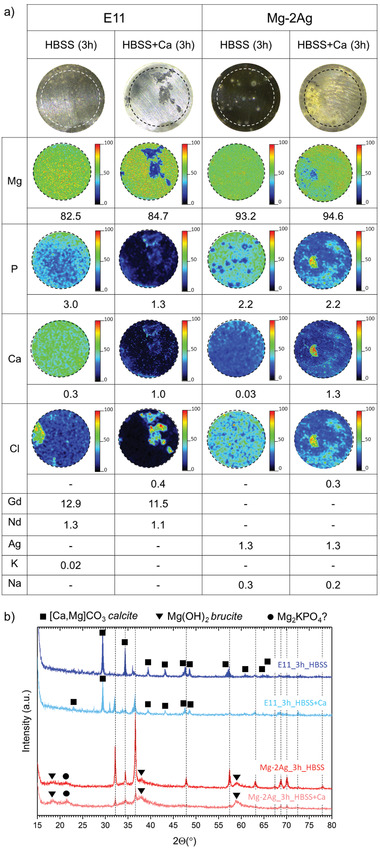
Composition of the degradation products layer generated on the E11 and Mg‐2Ag samples after 3 h of dynamic immersion tests in HBSS (solution I) and HBSS with the addition of Ca^2+^ cations (solution III). a) The relative chemical analysis in wt% and element distribution of the performed by µ‐XRF on a 0.7 cm diameter area of the sample (1.5 cm^2^), and b) the identification of the crystalline phases by grazing angle XRD (GAXRD).

The immersion of the E11 alloy on HBSS (solution I) composition revealed a nonhomogeneously corroded microstructure (**Figure** **5**a‐c). The high degradation degree of the primary *α*‐Mg dendrites revealed a cracked morphology of the degradation layer due to the drying process after immersion. Its chemical compositions revealed a high content of O and P but also Gd and Nd in higher concentrations than in the overall alloy composition, as presented in the EDX analysis, locations 1 and 3 of Figure 5m. Besides the apparently noncorroded IM particles and segregated *α*‐Mg zones, the EDX analysis 2 of Figure 5m revealed the presence of O and P at the degradation layer. In the case of the immersion of Mg‐2Ag, a homogeneous dark corroded surface under the optical microscope in Figure 5g revealed the less prone to the galvanic degradation microstructure of the extruded and solubilized Mg‐2Ag alloy. This lower degree of degradation is also evident in the still visible polishing marks of the surface preparation under the BSEM in Figure 5h,i. In the absence of any microstructural feature, the EDX analysis 5 of Figure 5m revealed an increase of O and P at the degradation layer. The presence of 0.3 wt% of Ca in the degradation products layer under immersion in HBSS is justified by the 0.19 wt% of Ca content in the E11 alloy. However, in the case of the Mg–2Ag, the low presence of Ca (0.03 wt%) in the degradation product layer revealed in **Figure** **6**a could be associated with; a) a Ca contamination in the flow circuit, or b) to residual values of Ca in the alloy, not detectable by μXRF, and coming from the primary magnesium production by the Pidgeon process using dolomite (Ca,Mg)CO_3_ as Mg source. And the consequent enrichment in the degradation products layer after the degradation process.

The influence of Ca^2+^ cations in the HBSS composition (solution II) on the E11 degradation morphology is presented in Figure 5d–f. Primary *α*‐Mg dendrites revealed a noncracked morphology with enrichment in Gd and O observed before but with an increase of the Ca content and a significantly decreased P relative proportion, as revealed by the EDX analysis 4 of Figure 5m. In the case of the extruded Mg–2Ag alloy, the immersion in HBSS with CaCl_2_·2H_2_O resulted in a bright surface of Figure 5j, revealing a general degradation process. The degradation products layer revealed areas of lower O, P, Ca and Ag content (EDX analysis 7 of Figure 5m) and other areas aligned in parallel to the polishing marks with higher degradation process presenting higher quantities of O, P, Ca and Ag (EDX analysis 6 of Figure 5m). Highlighted white arrows in Figure 5d‐j.

The presence of Ca^2+^ in the HBSS composition typically led to a breakdown of filiform corrosion. Nevertheless, this was accompanied by an active precipitation process, so that the corrosion rate of both alloys in HBSS+Ca^2+^ electrolyte was significantly lower than that in simple HBSS. The high density of those globular precipitates presented for the Mg–2Ag in the EDX analysis 8 of Figure 5m was characterized with a significant increase of O, Ca, and P compared to the relative composition of the rest of the areas, as presented for the Mg–2Ag. Those filiform corroded regions presented a Ca‐P co‐precipitation process characterized with a Ca/P ratio of 0.8 and 0.6 for the E11 and Mg–2Ag, respectively, besides a high Cl concentration, as was analyzed by μ‐XRF in Figure 6a.

Both alloys presented an accumulation of the main alloying elements in the degradation products layer. The Gd accumulation presented by the EDX analysis 1 and 3 of Figure 5m and corroborated by μ‐XRF in Figure 6a for that in the E11 alloy, points to a complex process that involves the preferential dissolution of the Mg matrix versus the stronger cathodic behavior of a) the pseudo‐eutectic G,Nd‐rich phase, possibly *β*‐Mg_5_(Gd,Nd),^[^ ^35^, ^47^ ^]^ b) the *α*‐Mg segregated on Gd and Nd surrounding the secondary phases, and c) the presence of [Gd,Nd,Mg]‐hydrides (REH_2_/REH_3_) phases.^[^ ^48^, ^49^ ^]^ The higher dissolution resistance of those phases promotes their inclusion in the degradation product layer. In addition to a corrosion/dealloying process, as presented for the intermetallic phase in the Mg–Zn–Ca system by Cihova et al.,^[^ ^50^ ^]^ this situation could promote the preferential dissolution of Mg from those phases and therefore contributing to the gradual enrichment of Gd. The accumulation of Ag in the degradation product layer of the Mg–2Ag alloy, presented in points 5 and 6 of Figure 5, could be related to the 0.5–1.5 nm star‐like bright precipitates homogeneously disseminated over the surface, as shown in Figure 5h,i. This precipitation can be associated with the AgCl precipitation revealed previously by XPS analysis^[^ ^51^ ^]^ due to its low solubility in water (*K*_sp_ (25 °C) = 1.8 × 10^–16^ mol^2^ cm^‐6 [^ ^52^ ^]^). The presence of phases containing alloying elements (e.g., AgCl) opens the question of their contribution to the surface's passivation. However, in the present study, the presence of Ag^+^ and Cl^–^ under both conditions (HBSS and HBSS with the addition of CaCl_2_·2H_2_O), justify the possible protective contribution of AgCl in both situations. Therefore, this effect should not mask the protective effect of the Ca‐containing phases.

The inhomogeneity of the as‐cast microstructure of the E11 alloy and a higher content of more noble elements (Gd, Nd) are responsible for the strong galvanic corrosion processes that promote significantly higher hydrogen evolution when compared to the more homogeneous Mg–2Ag alloy. This effect is visible for the 24 hours of immersion in both HBSS and HBSS with the presence of Ca^2+^, as revealed in Figure 3.

The degradation products layer was also characterized by grazing incidence X‐ray diffraction (GAXRD) with an incidence angle of *α* = 1° relative to the disc surface to reveal compositional characteristics of the surface, minimizing the bulk alloy signal. The analysis presented in Figure 6b showed differences between the crystalline compounds found on the E11 and the Mg–2Ag alloys, but with no significant compositional changes between the absence or presence of Ca^2+^ cations in the simulated physiological solution. This indicates that the crystalline phases detected are not responsible for the low interface pH in the presence of Ca^2+^ cations. The E11 surface generated under immersion in commercial HBSS revealed the presence of a *calcite* structure in which Mg atoms can be partially substituted by Ca atoms, [Ca,Mg]CO_3_. There was no apparent signal from the Mg structure, probably due to the low grazing angle and the degradation product layer thickness. On the contrary, the GAXRD analysis on the Mg–2Ag surface showed a strong presence of the Mg peaks revealing the lower thickness of the degradation products layer. Moreover, the presence of Mg(OH)_2_ brucite was identified. Besides, the peak at *θ* = 21.4° could be assigned to an Mg‐phosphate with a stoichiometry of Mg_2_KPO_4_. However, as there was no significant signal from K under immersion in HBSS, identifying this compound by only one diffraction peak would not assure this compound's presence. With the addition of CaCl_2_·2H_2_O to the HBSS composition (solution II), the relative intensity of the [Ca,Mg]CO_3_ diffractions peaks on the E11 and the Mg signals on the Mg–2Ag show a decrease in the intensity. In contrast, the relative intensity of the Mg(OH)_2_ *brucite* diffractions peaks for the degradation layer on Mg–2Ag seems to increase.

**Figure 7:**
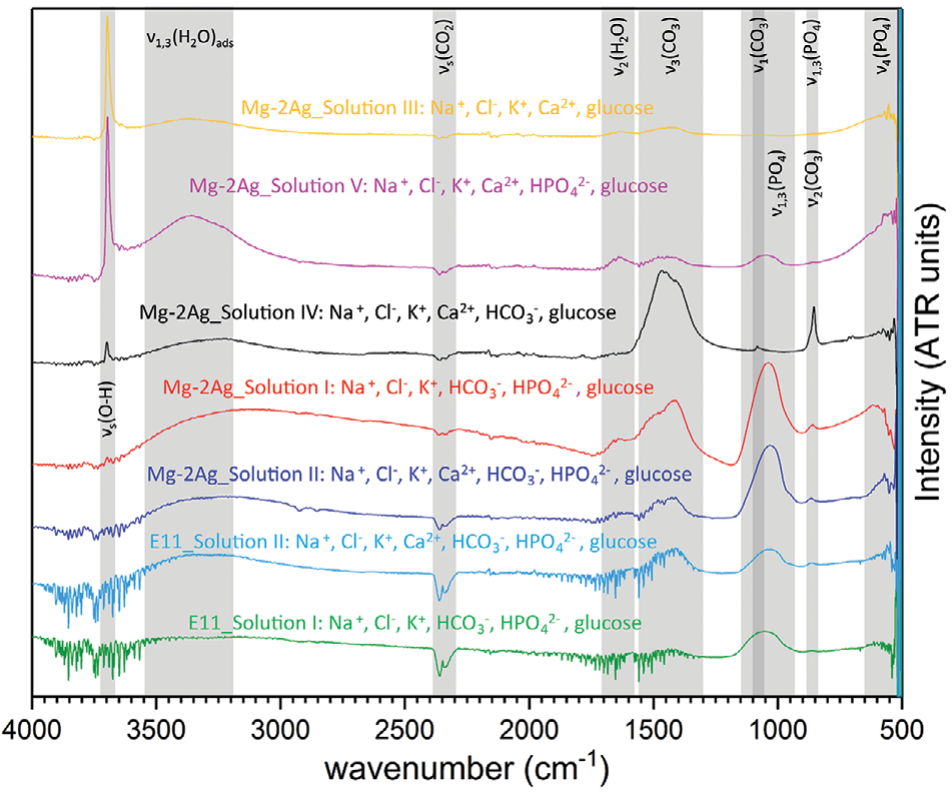
Influence of the different inorganic components of the simulated physiological solution on the composition of the outer part of the degradation products layer analyzed by FTIR‐ATR on the corroded Mg‐2Ag surface after 3 h of dynamic immersion (1.5 mL min^‐1^) in the following solutions: III (Na^+^, Cl^–^, K^+^, Ca^2+^ and glucose), solution V (Na^+^, Cl^–^, K^+^, Ca^2+^, HPO_4_^2–^ and glucose), solution IV (Na^+^, Cl^–^, K^+^, Ca^2+^, HCO_3_^–^ and glucose), solution I (Na^+^, Cl^–^, K^+^, HCO_3_^–^, HPO_4_^2–^ and glucose), and solution II (Na^+^, Cl^–^, K^+^, Ca^2+^, HCO_3_^–^, HPO_4_^2–^ and glucose). The influence of the presence of the Ca^2+^ cations in the HBSS composition on the E11 and Mg–2Ag was inferred by comparison of the spectra generated after 3 h of dynamic immersion in solution I and solution III for both alloys.

Since conventional X‐ray diffraction techniques cannot detect the presence of amorphous or even nanocrystalline phases, the degradation products layer was also studied by FTIR‐ATR, which results are depicted in **Figure** **7**. Due to the presence of glucose in the HBSS commercial composition (solution I, Table 1), this component was added in the same concentration to all simulated physiological solutions. Considering the equal contribution of the glucose in the IR signal for all the formed degradation products layers, the analyzed IR spectra revealed information about the inorganic polyatomic ions present in the degradation products layer.

The yellow spectrum presents the IR signal of the degradation products layer formed under immersion of the Mg–2Ag surface in the presence of Na^+^, Cl^–^, K^+^, Ca^2+^ ions and glucose according to the composition of the solution III presented in Table 1. This spectrum shows an intense and sharp band at 3696 cm^–1^ that might be associated with the O—H bonds present in glucose molecules but can also result from the contribution of the *A*_2u_ infrared active mode of Mg(OH)_2_.^[^ ^53^, ^54^ ^]^ The lower relative intensity of the $\nu_{3}\left({\mathrm{CO}}_{3}^{2-}\right)$ and $\nu_{2}\left({\mathrm{CO}}_{3}^{2-}\right)$ bands might be related to the presence of the glucose and the reaction between the CO_2_ dissolved in the simulated physiological solution and the Mg^2+^ and Ca^2+^ cations that promote the precipitation of Mg, Ca‐carbonates on the surface. The black spectrum was obtained under immersion in the solution IV (with the presence of Na^+^, K^+^, Cl^–^, Ca^2+^, HCO_3_^–^ ions and glucose) according to Table 1. The increment of HCO_3_^–^ in the solution revealed an increment in the intensity of the $\nu_{3}\left({\mathrm{CO}}_{3}^{2-}\right)$ band at 1462 cm^–1^, as well as the presence of the low intensity $\nu_{1}\left({\mathrm{CO}}_{3}^{2-}\right)$ and the sharp $\nu_{2}\left({\mathrm{CO}}_{3}^{2-}\right)$ modes at 1083 cm^–1^, at 854 cm^–1^ respectively. Those bands have been previously identified as characteristic bands belonging to the vibrational modes of the carbonate molecule in amorphous and anhydrous MgCO_3._^[^ ^55^ ^]^ Moreover, the less relative intensity of the sharp band at 3696 cm^–1^ in comparison with the $\left({\mathrm{CO}}_{3}^{2-}\right)$ corresponding bands, points to a higher relevance of the of Mg, Ca‐carbonates in comparison to the Mg(OH)_2_ phase, on the outer part of the degradation products layer. The pink spectrum was revealed by the degradation products layer generated under immersion in the solution V with the presence of Na^+^, Cl^–^, K^+^, Ca^2+^, HPO_4_^2–^ and glucose. In contrast to the previous spectrums, a broadband appears around 1033 cm^–1^ corresponding to the combination of the $\nu_{3}\left({\mathrm{PO}}_{4}^{3-}\right)$ and $\nu_{1}\left({\mathrm{PO}}_{4}^{3-}\right)$ modes, as described in ref. [56, 57, 58, 59]. Similar relative intensity to the ${\mathrm{PO}}_{4}^{3-}$ modes is presented by the corresponding $\nu_{3}\left({\mathrm{CO}}_{3}^{2-}\right)$ and $\nu_{2}\left({\mathrm{CO}}_{3}^{2-}\right)$ bands, presumably by the contribution of the HCO_3_^–^ ions coming form the atmospheric CO_2_ dissolved in the solution. All mentioned bands appear with considerably lower relative intensity compared with the sharp *ν*(O—H) band at 3696 cm^–1^.

**Figure 8:**
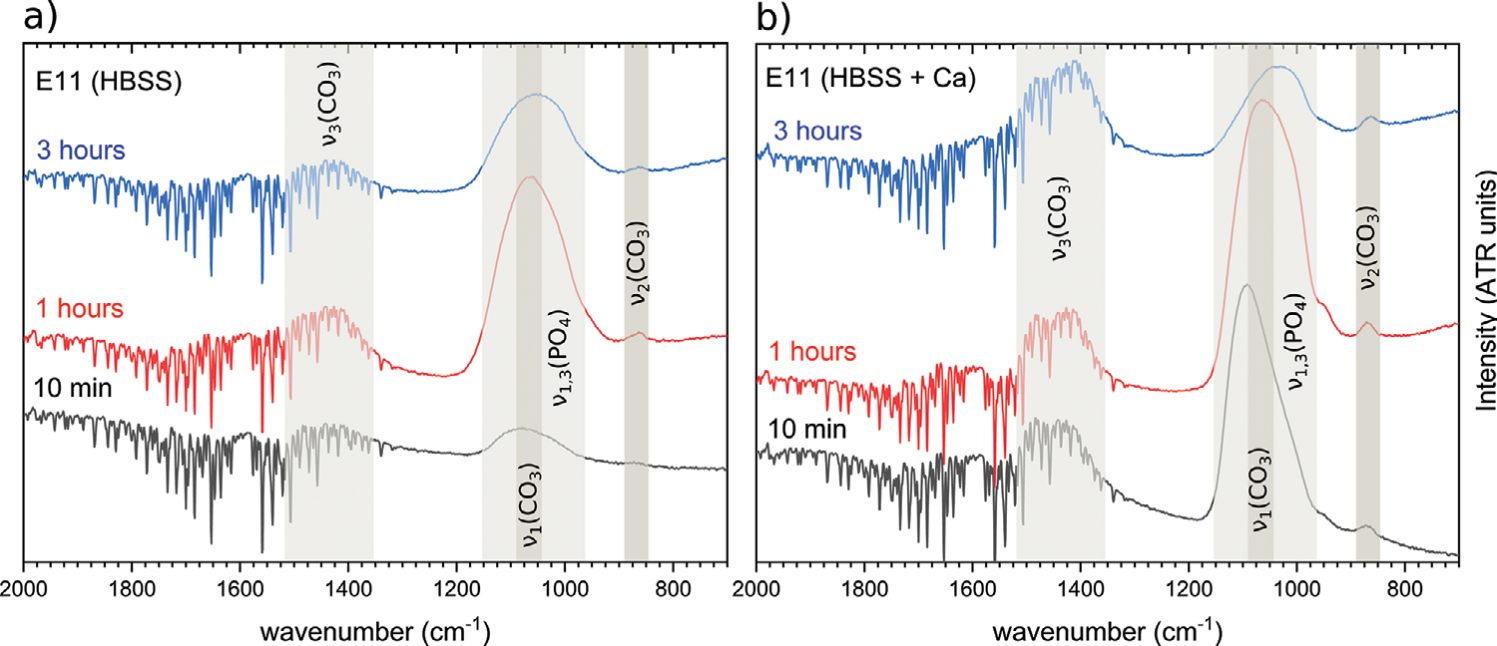
Evolution of the carbonates and phosphates IR absorption bands by FTIR‐ATR on the degradation products layer formed after 10 min, 1, and 3 h of dynamic immersion (1.5 mL min^‐1^). The immersion tests were performed on as‐cast E11 samples under a) commercial HBSS (solution I) and b) commercial HBSS with the addition of 2.5 × 10^‐3^ m CaCl_2_·2H_2_O (solution III).

The subsequent analysis of the degradation products layer generated on E11 and Mg–2Ag alloys after 3 h of immersion in commercial HBSS and commercial HBSS in the presence of 2.5 × 10^‐3^ m Ca^2+^ ions is presented in the bottom part of Figure 7. Under both immersion conditions, the same absorption bands could be identified for both alloys. However, in the case of the Mg–2Ag alloy, an increase in relative intensity of the $\nu_{3}\left({\mathrm{PO}}_{4}^{3-}\right)$ concerning the $\nu_{3}\left({\mathrm{CO}}_{3}^{2-}\right)$ absorption band was visible when the Ca^2+^ cations were present (solution II, blue spectrum Figure 7) in contrast with a higher contribution of carbonates revealed by a higher relative intensity of the $\nu_{3}\left({\mathrm{CO}}_{3}^{2-}\right)$ absorption band in the absence of Ca^2+^ cations (solution I, red spectrum Figure 7). This fact points to a higher presence of phosphates, possibly Ap‐CaP phases, in the outer layer promoted by the presence of the Ca^2+^ cations. On the contrary, the study of the Ca^2+^ contribution on the E11 alloy revealed the opposite situation since the presence of Ca^2+^ cations (solution II, light blue spectrum Figure 7) generated a higher intensity of the $\nu_{3}\left({\mathrm{CO}}_{3}^{2-}\right)$ band with respect to the $\nu_{3}\left({\mathrm{PO}}_{4}^{3-}\right)$ band. According to these results, the degradation products layer seems to be formed by different precipitation processes in the case of the E11 and the Mg–2Ag (T4) alloys. This might be related to the different degradation rate presented by both alloys in Figure 3c; however, both surfaces presented the same low interface pH, as was shown in Figure 3a,b. The evolution of the relative intensity of the $\nu_{3}\left({\mathrm{PO}}_{4}^{3-}\right)$ and the $\nu_{3}\left({\mathrm{CO}}_{3}^{2-}\right)$ bands over the immersion time was studied on the degradation products layers generated after 10 min, 1 h and 3 h on the E11 surface immersed in HBSS (solution I) and HBSS with the addition of CaCl_2_·2H_2_O (solution II), as shown in **Figure** **8**.

The immersion of the E11 alloy in HBSS shows an initial precipitation process based on the same contribution of the Mg‐carbonates and Mg‐phosphates revealed by the same relative intensity of the $\nu_{3}\left({\mathrm{PO}}_{4}^{3-}\right)$ and the $\nu_{3}\left({\mathrm{CO}}_{3}^{2-}\right)$ bands. After at least 1 h of dynamic immersion follows an Mg‐phosphate dominant precipitation process that continues at least until 3 hours of immersion, according to the increment of the $\nu_{3}\left({\mathrm{PO}}_{4}^{3-}\right)$ intensity concerning the $\nu_{3}\left({\mathrm{CO}}_{3}^{2-}\right)$ band presented in Figure 8a. On the other hand, the presence of Ca^2+^ cations, Figure 8b, the higher relative intensity of the $\nu_{3}\left(PO_{4}^{3-}\right)$ band after 10 minutes of immersion revealed fast initial dominant Ca, Mg‐phosphates precipitation that lasts until at least 1 h of immersion. However, after 3 h of immersion, the relative intensities of both bands $\nu_{3}\left({\mathrm{PO}}_{4}^{3-}\right)$ and $\nu_{3}\left({\mathrm{CO}}_{3}^{2-}\right)$ are equal, pointing to an increase of the Ca, Mg‐carbonates precipitation process.

## 4 Discussion

### 4.1 Interface pH versus Dissolution Kinetics

According to the electrochemical reactions involving Mg dissolution in aqueous electrolytes, the reduction reactions of water molecules (Equation 2) and the dissolved oxygen (Equation 3)^[^ ^60^ ^]^ contribute to the release of OH^–^ ions and, therefore the evolution of the pH is related to the dissolution rate of the magnesium alloy.

(1) $$\mathrm{Mg}\to Mg^{2+}+2e^{-}$$

(2) $$2H_{2}O+2e^{-}\to H_{2}+2OH^{-}$$

(3) $$1/2O_{2}+H_{2}O+2e^{-}\to 2OH^{-}$$

As depicted in Figure 3, the E11 and Mg–2Ag alloys presented significantly different mass loss after 24 h of immersion. However, similar interface pH values were developed for both alloys for at least 24 hours of immersion, according to our previous work.^[^ ^27^ ^]^ These results contradict the possibility of comparing different alloys’ performance by direct correlation with changes in the bulk solution pH happening during the degradation process as presented in previous works.^[^ ^37^, ^61^ ^]^ This can be explained by the double effect of the degradation products layer at the interface environment: i) the physical barrier effect and ii) the localized buffering effect at the interface material/solution. The degradation products layer acts as a barrier against the diffusion of the products and reactants (e.g., H_2_O and OH^–^ ions) involved in the electrochemical processes of magnesium degradation. Therefore, it modulates the dissolution kinetics of the alloy. This modulation effect depends on the compactness and thickness of the degradation products layer. However, as shown in the mass loss values presented in Figure 3, this barrier effect, even with the protective degradation layer promoted by the presence of Ca^2+^ cations, cannot promote similar degradation rates for both E11 and Mg–2Ag alloys.

On the other hand, the base characteristic of the anions involved in the phases present in the degradation product layer (e.g., CO_3_^2–^, PO_4_^2–^) generates secondary acid‐base reactions that confer buffering capacities to the precipitation‐dissolution processes generated at the interface. In contrast to the barrier modulating effect to the dissolution kinetics exposed before, the pH buffering effect generated by the precipitation/dissolution processes of the degradation products layer is capable of absorbing high releasing rates of OH^–^ coming from high degradation kinetics. This is why Figure 3 shows a similar interface pH developed for two alloys with considerable differences in the mass loss under immersion in HBSS and HBSS with the addition of CaCl_2_·2H_2_O.

### 4.2 Interface Environment and Mg Degradation Mechanism

**Figure 9:**
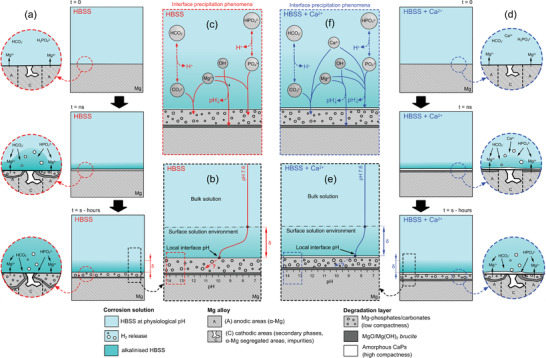
Schematic representation of the degradation mechanism of Mg‐based materials under immersion under HBSS in the absence of Ca^2+^ cations (in red) and in the presence of Ca^2+^ cations (in blue): a) Development of the degradation layer over the immersion time in HBSS, b) pH profile developed over the Mg surface under dynamic conditions in HBSS, c) heterogeneous precipitation phenomena of the inorganic phases involved in the formation of the degradation products layer in HBSS that are the source of the interface pH stabilization, d) development of the degradation layer over the immersion time in HBSS in the presence of Ca^2+^ cations, e) pH profile developed over the Mg surface under dynamic conditions in HBSS in the presence of Ca^2+^ cations, f) heterogeneous precipitation phenomena of the inorganic phases involved in the formation of the degradation products layer in HBSS in the presence of Ca^2+^ cations, that are the source of the interface pH stabilization. The question mark and the dashed lines denote the unknown pH microenvironment developed along with the degradation products layer. (*δ* identifies the thickness of the diffusion layer bordering the bulk solution).

**Figure 10:**
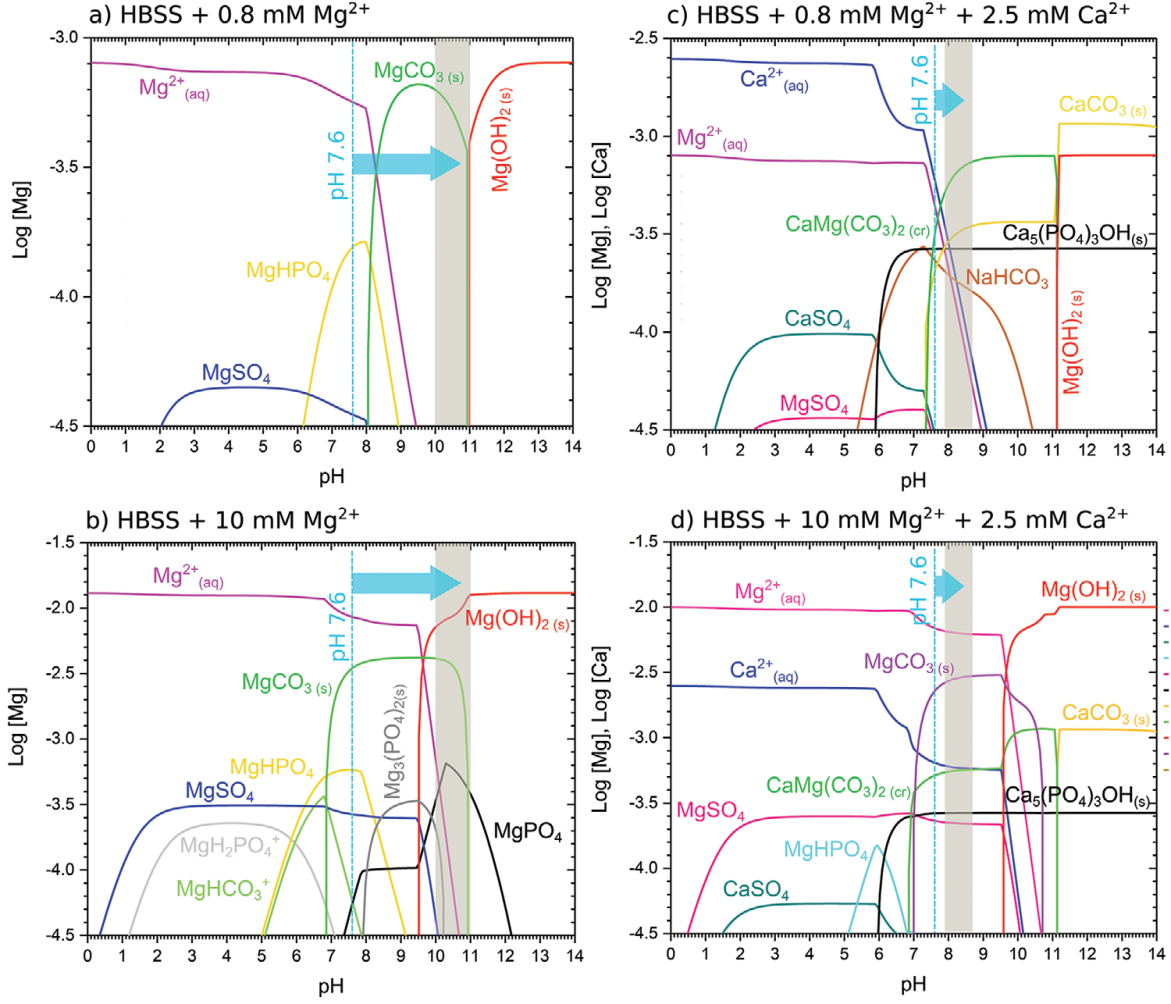
Concentration (m) in logarithmic scale (log [Mg^2+^], log[Ca^2+^]) for the predicted phases present under the estimated concentrations and measured interface pH for an Mg surface immersed in a solution with a composition of a) HBSS in the presence of 0.8 × 10^‐3^ m Mg^2+^ cations, b) HBSS in the presence of 10 × 10^‐3^ m Mg^2+^ cations, c) HBSS with the addition of CaCl_2_·2H_2_O in the presence of 0.8 × 10^‐3^ m Mg^2+^ cations, and d) HBSS with the addition of CaCl_2_·2H_2_O in the presence of 10 × 10^‐3^ m Mg^2+^ cations. The dashed line presents the initial value of the degradation solution pH, and the grey band presents the interface pH measured under HBSS with the addition of Ca^2+^.

Under immersion in the presence of Mg^2+^, HCO_3_^–^ and HPO_4_^2–^ ions, Mg alloys develop a fast initial alkalinization promoting the formation of a thin bilayer structure based on MgO/ Mg(OH)_2_ brucite. This thin layer partially passivates the surface of the alloy and modulates the degradation rate. Since a saturated solution in brucite, *K*_sp_ (25 °C) of 5.61 × 10^–12^,^[^ ^62^ ^]^ will promote a pH of 10.35, an interface pH of 10–10.5 units, found in Figure 3 for the E11 and Mg‐2Ag immersed in HBSS is ruled by the dissolution/precipitation process of the Mg(OH)_2_ brucite. This is probably why the immersion of Mg alloys in simple NaCl solutions generates similar interface pH^[^ ^28^ ^]^ than in HBSS in the absence of Ca^2+^ cations. According to **Figure** **9**a, this pH range stabilizes the precipitation of the second layer of Mg‐carbonates/phosphates, as can be interfered from the diagrams presented in **Figure** **10**a,b. The presence of these Mg‐carbonates/phosphates promotes a microenvironment underneath with possibly higher concentrations of Mg^2+^. This microenvironment can sift the stability of the Mg(OH)_2_ brucite to lower pH values, and therefore justify the presence of this phase after several hours of immersion of the Mg–2Ag alloy in Figure 6b. This low protective layer structure is responsible for the higher degradation rates presented for the E11 and the Mg–2Ag samples in Figure 3 under immersion in HBSS. This is reflected at the microstructure level with the aggressive dissolution of the *α*‐Mg matrix phase present in the E11 observed in Figure 5b,c.

On the contrary, under immersion in HBSS with the addition of CaCl_2_·2H_2_O, the presence of Ca^2+^, HCO_3_^–^ and HPO_4_^2–^ ions result in the rapid formation^[^ ^27^ ^]^ of a thin but compact outer layer on the Mg surface.^[^ ^6^ ^]^ The protective effect associated with its compactness was revealed to persist over the immersion time according to previous work^[^ ^50^ ^]^ and is corroborated in the present work by the EIS measurements of Figure 4. Moreover, this protective effect is also reflected in the filiform degradation processes presented in Figure 5d–j, which only happened when both alloys were immersed in the presence of Ca^2+^ cations. The composition of this layer is based on poorly crystalline or amorphous Ap‐CaP phases, as is supported by the Ca,P‐coprecipitation revealed by μ‐XRF in Figure 6a, and the higher contribution of phosphates presented in Figure 8 within the first 10 minutes of immersion among carbonates and Mg(OH)_2_ brucite. After the development of the thin Ap‐CaP layer, the corrosion front proceeds inwards the Mg alloy, as shown by Cihova et al.^[^ ^50^ ^]^. This condition transforms, at least, the thermodynamic conditions (ionic activities and pH) under between the Ap‐CaP layer and the metallic surface, and generates different degradation products, as described in Figure 9d–e. Under this hypothetical high pH and Mg^2+^ concentration, Figure 10d predicts a decrease in the HA phase, CaCO_3_ and the Ca,Mg(CO_3_)_2(cr)_ fractions, and the increase of the MgCO_3_ fraction. Moreover, such microenvironment will also stabilize the presence of Mg(OH)_2_ brucite phase, confirmed by GAXRD in Figure 6. This increase in the MgCO_3_ fraction precipitation might be the reason for the decrease in the relative intensity of the $\nu_{3}\left({\mathrm{PO}}_{4}^{3-}\right)$ band with respect to the $\nu_{3}\left({\mathrm{CO}}_{3}^{2-}\right)$ band over the immersion time exposed in Figure 8. Accordingly to above, the protective effect presented in Figure 3 for the E11 and Mg–2Ag samples under immersion in HBSS in the presence of Ca^2+^ cations corresponds to the multilayer structure composed by a compact but not fully covering inner Mg/Mg(OH)_2_ layer, an intermediate porous Mg‐carbonate layer, and a thin and compact Ap‐CaP outer layer as presented in Figure 9d–f.

Under the presence of Ca^2+^ cations, the precipitation of several Ca‐containing phases like HA (Ca_5_(PO_4_)_3_OH), CaMg(CO_3_)_2_, and CaCO_3_ depicted in Figure 10c,d, buffer the interface pH in a range of 7.8–8.5 units. It should be mentioned that more realistic phases like Mg^2+^ and CO_3_^2–^ partially substituted hydroxyapatite (Ca_a_Mg_b_(PO_4_ or CO_3_)_c_(OH)_d_), found under in vivo^[^ ^63^ ^]^ and under in vitro conditions,^[^ ^64^, ^65^ ^]^ could not be included in the thermodynamic prediction of Figure 10 because neither their stoichiometry nor the stability constants are known.

### 4.3 Relevance of the Results for Future Investigations and Material Design

According to the discussion above, Mg‐based materials present a stable interface pH of about 8–8.5 units in the presence of Ca^2+^, HPO_4_^2–^ and HCO_3_^–^ ions. Even though those results are not conclusive, due to the lack in the simulation of the physiological conditions presented in this work, statements like the negative effect in the osteointegration presented by Song et al.^[^ ^66^ ^]^ or the positive stimulation of the callus formation effect proposed by Zhao et al.^[^ ^67^ ^]^ promoted by a highly alkaline environment at Mg surface, should be questioned. In order to confirm or refute the results presented here, progress will need to be made in; a) defining the targeted implantation site environment (e.g., ions concentration evolution, physiological fluid exchange), and b) clarifying the individual and synergic influences of other physiological components like the organic molecules and cell presence.

As described in the previous section, the dissolution of Mg‐based materials is strongly influenced by the formation of the degradation products layer on the surface (number of phases precipitated, their composition, and the compactness of the layer controls), and this, in turn, is influenced by the local degradation environment. The local degradation environment is also dependent on the mass transfer between both environments and depends on the testing set‐up applied (e.g., static, dynamic, stirring, buffering system). Since a concentration profile was found in the present work for a high diffusivity species as the OH^–^ ion (*D*_OH_^–^ = 5.260 × 10^–9^ m^2^ s^–1^), similar concentration profiles might be assumed, but in a different extension, for slower diffusivity species like Mg^2+^ (*D*_Mg_^2+^ = 0.7063 × 10^–9^ m^2^ s^–1^), Ca^2+^ (*D*_Ca_^2+^ = 0.7914 × 10^–9^ m^2^ s^–1^), or HCO_3_^–^ (*D*_HCO3_^–^ = 1.105 × 10^–9^ m^2^ s^–1^) ions,^[^ ^68^ ^]^ or for the species like HPO_4_^2–^ (*D*_HPO4_^2–^ = 8.8 × 1^–10^ m^2^ s^–1^), or PO_4_^3–^ (*D*_PO4_^3–^ = 5.8 × 10^–10^ cm^2^ s^–1^).^[^ ^69^ ^]^ Therefore, the knowledge of the evolution of ionic activities at the interface, including the pH, for different magnesium‐based systems and different fluid exchange rates, and the design of new set‐ups or techniques to measure them with a good compromise of spatial and time resolution, is of crucial importance to understand the chemical processes of Mg degradation under simulated physiological conditions. Moreover, this knowledge will help to study the composition of Mg and other degradable biomaterials including coating systems that help to stabilise the interface pH and protective degradation products layers under different implantation site environments.

## 5 Conclusions

This study contributes to defining the chemical surface processes happening during Mg degradation under simulated physiological conditions. The evolution of the bulk pH and interface pH by scanning ion‐selective electrode technique (SIET) on E11, and Mg–2Ag, were monitored and correlated with the composition of the degradation products layer, leading to the following findings:

- The precipitation/dissolution processes of the inorganic phases in the degradation products layer generate an extra pH buffering system at the surface. This buffering system can absorb different alkalinization rates promoted by Mg‐based materials with significant differences in the degradation rate, stabilizing the value of the interface pH
- The immersion of Mg–2Ag and E11 alloys in HBSS under flow conditions (1.5 mL min^‐1^), generates a non‐protective degradation products layer based on Mg(OH)_2_ and Mg‐phosphates/carbonates that promotes an interface pH between 9.8 and 10.2.
- The immersion of Mg–2Ag and E11 alloys in HBSS containing Ca^2+^ cations generates the protective precipitation of Mg(OH)_2_ and Ap‐CaP that stabilizes the interface's pH between 7.6 and 8.0. The protective Ap‐CaP layer hinders the mass transfer between the simulated physiological solution and the metallic surface, and the growth of the degradation products layer continues with the formation of an intermediate Mg‐carbonates layer. The synergic protective effect between all the degradation products layers slows down significantly the degradation rate.

## Conflict of Interest

The authors declare no conflict of interest.

## Author Contributions

J.G: Conceptualization of the manuscript, characterization analysis, preparation of the manuscript, including the data visualization. S.V.L: Supervision, the conceptualization of the manuscript and manuscript revision. D.M: Characterization analysis and manuscript revision. N.S: Characterization analysis and manuscript revision. F.F: manuscript revision, M.L.Z: manuscript revision. R.W.‐R: manuscript revision.

## Data Availability

Data available on request from the authors.
